# A Rare Case of Caecum Perforation Secondary to Campylobacter jejuni Infection

**DOI:** 10.7759/cureus.65565

**Published:** 2024-07-28

**Authors:** Wessam Al Dallal, Hassan Jouni, Mohamed Wael Ahmed, Ali Yasen Mohamedahmed, Georgios Kakaniaris

**Affiliations:** 1 General Surgery, Queen's Hospital Burton, University Hospitals of Derby and Burton NHS Foundation Trust, Burton on Trent, GBR; 2 General and Colorectal Surgery, Queen's Hospital Burton, University Hospitals of Derby and Burton NHS Foundation Trust, Burton on Trent, GBR

**Keywords:** infectious colitis, caecum perforation, gastroenteritis, large bowel perforation, campylobacter jejuni

## Abstract

Campylobacter is a common cause of bacterial gastroenteritis worldwide. This report presents a rare case of a 44-year-old female who developed a caecum perforation following an initial diagnosis of *Campylobacter jejuni* infection. The patient initially presented with several episodes of diarrhoea, vomiting, and abdominal pain. Initial investigations confirmed an uncomplicated *C. jejuni* infection, which was treated with antibiotics and supportive measures. Despite treatment, the patient's symptoms persisted and worsened, and caecum perforation was confirmed on the abdomen and pelvis computed tomography. The patient underwent an emergency right hemicolectomy with an end ileostomy and was discharged home on postoperative day 14 after she made a full recovery. Healthcare professionals should be vigilant for possible severe complications in patients with *C. jejuni* infection. Frequent abdominal examinations with radiological investigations should be considered when symptoms are worsening to promptly identify any potentially life-threatening complications similar to those in the presenting case.

## Introduction

*Campylobacter jejuni* is a leading cause of infectious gastroenteritis worldwide, which usually requires conservative management with only a small number of cases necessitating hospitalisation [1]. In rare instances, acute enteric complications could be encountered, including mesenteric adenitis, pseudomembranous colitis, massive gastrointestinal bleeding, ischemic bowel disease, and bowel perforation [2]. Bowel perforations caused by *C. jejuni *infections are extremely uncommon and are often preceded by a toxic megacolon [3]. This case report presents a rare pancolitis caused by *C. jejuni*, leading to ascending colon perforation with a retroperitoneal abscess without a toxic megacolon; only a handful of similar cases have been reported in the literature. Moreover, a literature review of similar cases has been included. 

## Case presentation

A 44-year-old female presented to the emergency department with a three-day history of right-sided abdominal pain associated with 15 episodes of bloody diarrhoea with mucous per day and multiple episodes of vomiting. She denied significant past medical diseases or recent travel history. Initial abdominal examination showed rebound tenderness in the right iliac fossa. Stool cultures returned positive for a *C. jejuni* infection and faecal calprotectin was negative. Moreover, blood test investigations showed a white cell count (WCC) of 15.6x10^9^/L (normal range, 4.5-11.0×10^9^/L) and a C-reactive protein (CRP) of 260 mg/L (normal range ≤ 1 mg/L). The rest of the blood investigations were normal. Computed tomography of the abdomen and pelvis (CTAP) findings were consistent with severe pancolitis. The patient was treated for gastroenteritis with intravenous Tazocin, intravenous fluids, and analgesia. 

**Figure 1:**
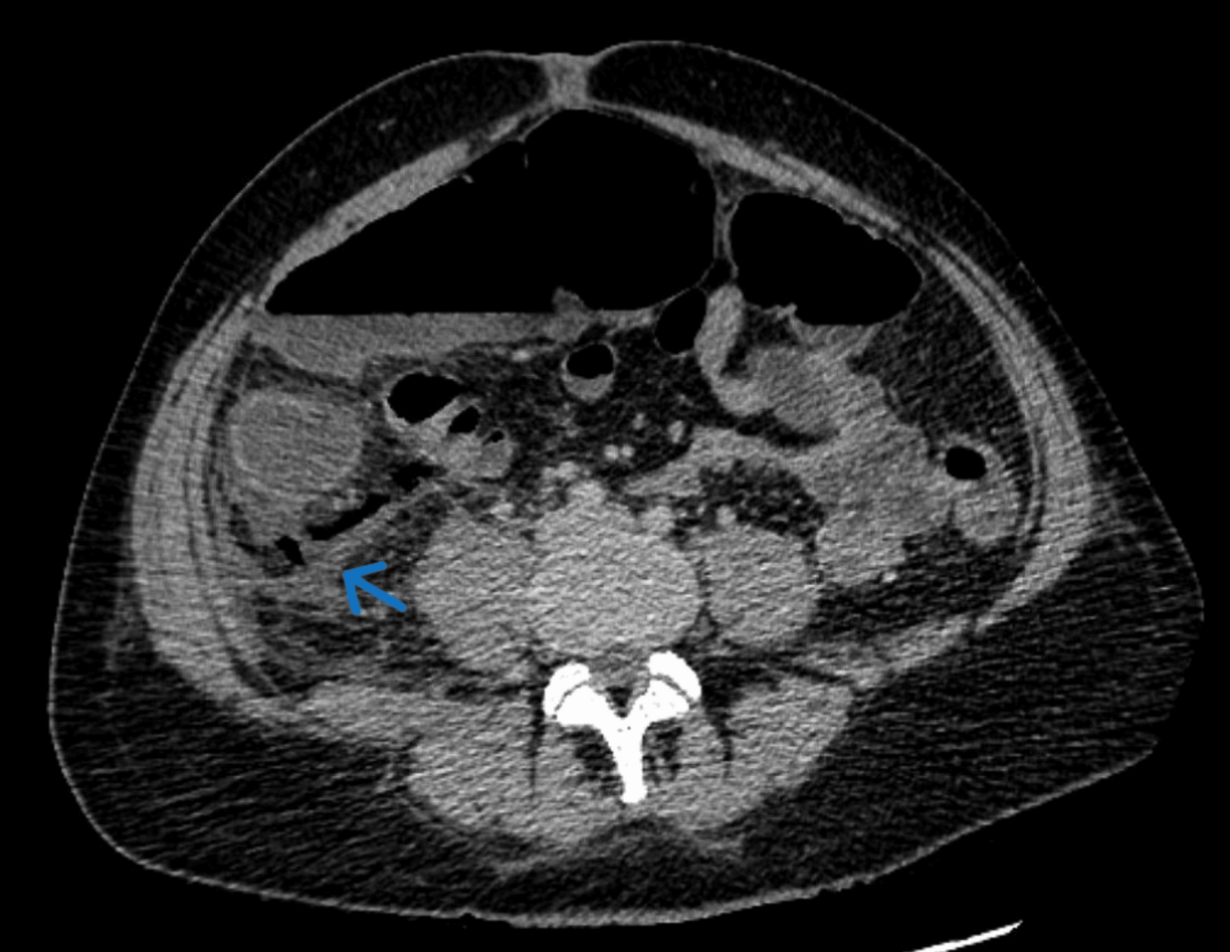
CT abdomen and pelvis (axial section) demonstrating free air and fluids in the right side of the abdomen (blue arrow)

**Figure 2:**
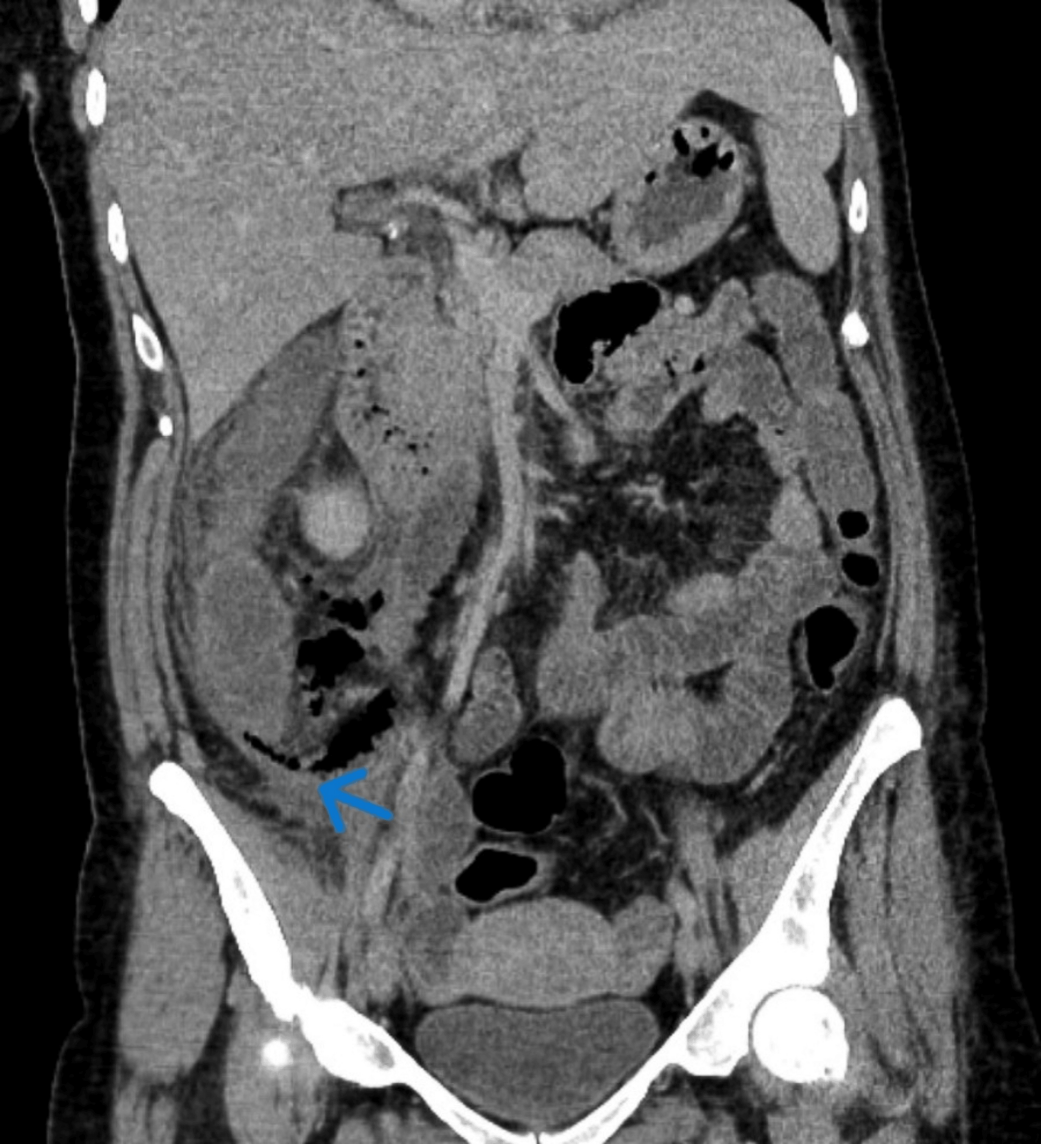
CT abdomen and pelvis (coronal section) demonstrating free air and fluids in the right side of the abdomen (blue arrow).

On day 2 of admission, the patient's symptoms had not resolved. Abdominal examination revealed guarding, diffuse rebound tenderness, and pain with slight movement. A venous blood gas was normal apart from lactate of 4.0 mmol/L (normal range, 0.5-1 mmol/L). A repeat CTAP confirmed a caecal perforation with faecal material in the right paracolic gutter (Figures 1, 2).

Consequently, the patient underwent an emergency open right hemicolectomy with an end ileostomy. Operative findings were a posterior perforation at the junction of the caecum and the proximal ascending colon with a retroperitoneal abscess containing pus with feculent contamination. Furthermore, there were ischemic patches over the caecum and proximal ascending colon. A 20-French (Fr) Robinson drain was inserted within the right paracolic gutter in addition to a 24-Fr rectal catheter.

The patient was admitted to the intensive care unit for monitoring for one day postoperatively. However, on postoperative day (POD) 7, the patient developed abdominal collection in the right subhepatic area, which was treated successfully with drainage under ultrasound guidance. *Streptococcus milleri* was isolated from the collection fluid, and co-amoxiclav and metronidazole were prescribed accordingly. The patient made a good recovery and was discharged home on POD 14. Histopathology of the hemicolectomy specimen showed features of inflammation with spots of necrotic patches. 

## Discussion

*C. jejuni* is one of the most common bacterial pathogens causing acute gastroenteritis alongside non-typhoidal *Salmonella*, Shiga toxin-producing *Escherichia coli*, and *Clostridium perfrinegens* [1,2]. A study conducted by Fleckenstein et al. in 2021 revealed that up to 1.5 million cases of *C. jejuni* infection are accounted for in the United States, leading to 8500 inpatient hospitalisations and 80 deaths [4]. In the United Kingdom alone, 500,000 cases of *C. jejuni* are encountered annually, with 80,000 requiring GP (general practitioner) consultations [5].

*C. jejuni *invades the jejunum and ilium and can extend into the large intestine, causing an inflammatory exudative state to the bowel called edematous enteritis [6]. It has an incubation period of approximately one week, usually followed by a one- to three-day prodromal stage where individuals experience fevers, rigours, dizziness, and malaise. This is typically followed by an acute diarrheal phase whereby the patient might experience 10 or more daily bowel motions associated with blood and mucous. Furthermore, patients commonly present with associated abdominal pain and occasional vomiting. These symptoms are usually self-limiting and resolve within five to seven days after the diarrheal phase, with the focus of treatment being hydration and electrolyte replenishment [7].

Antibiotics are typically reserved for severe cases that involve bloody diarrhoea, severe abdominal pain, and fever. This was especially relevant in the presented case, whereby the infection was severe and resulted in pancolitis and a caecal perforation. The stool culture isolated from the patient revealed a *C. jejuni* growth sensitive to erythromycin and resistant to fluoroquinolones such as ciprofloxacin. However, in the current case, the initial antibiotic used before receiving stool sensitivity results was intravenous Tazocin due to the broad spectrum cover it exhibits. Nevertheless, this was unlikely to prevent the preceding outcome of pancolitis.

Pancolitis is a very rare intestinal complication of *C. jejuni*, which in turn could lead to an intestinal perforation warranting surgical intervention, as seen in the current case. In this case, surgical findings revealed ischemic patches of bowel in the caecum and the ascending colon, the territories supplied by the superior mesenteric artery. This indicates that the patient has likely developed the caecal perforation secondary to non-occlusive mesenteric ischemia (i.e., ischemic colitis), a known but rare complication of infectious colitis [8].

However, perforations secondary to *C. jejuni* are usually preceded by colonic dilatation in the form of toxic megacolon [2,3]; this was not the case with the current patient. Initial and repeat CT findings in this patient showed no evidence of bowel luminal dilation, loss of haustral markings, or pseudo polyposis suggestive of toxic megacolon. Toxic megacolon is a life-threatening condition that involves acute dilatation of the colon associated with systemic toxicity [6]. This is a rare but devastating complication of infectious colitis with a mortality rate of 19%; the rate rises to 41% when accompanied by a bowel perforation in cases where the patient has progressive colonic dilatation with clinical deterioration, uncontrolled bleeding, or perforation despite optimised medical management addressing the underlying cause, and urgent surgical intervention is indicated [9,10].

Table 1 includes the summary of a literature review of 13 similar reported cases [2,3,11-20] with spontaneous perforation secondary to *C. jejuni *infection. It is worth mentioning that eight out of the 13 reported patients developed toxic megacolon preceding the perforation. Moreover, the caecum is the most common perforation site, constituting six of the 13 cases, similar to the present case. The second most common perforation site is the sigmoid colon, a watershed area susceptible to ischemia and supplied by the inferior mesenteric artery, known as Sudeck’s point [2,10]. Interestingly, Kundoly and Jolly reported a case with a small bowel obstruction in the distal ilium accompanied by the perforation secondary to the *C. jejuni* infection [20]. 

**Table 1:** Summary of reported cases with bowel perforation secondary to Campylobacter jejuni Infection. NA: not available

Case report and year	Age (years), Gender	Perforation site	Additional features
Vyas et al. (1993) [11]	38, Male	Caecum and sigmoid colon	Toxic megacolon
Larvol et al. (1994) [12]	38, Female	Transverse colon	Toxic megacolon
Kummar and Meyenberger (1998) [13]	53, Male	NA	Toxic megacolon
Jackson et al. (1999) [14]	50, Female	NA	Toxic megacolon, Crohn's disease
Cooke (1999) [15]	24, Female	Caecum	NA
Fang et al. (2000) [16]	5, Male	Caecum	Appendicitis and toxic megacolon
Fang et al. (2000) [16]	3, Male	Sigmoid colon	Toxic megacolon
Jassim et al. (2011) [17]	80, Female	Terminal Ilium	No toxic megacolon
Brantzen and Brantzen (2012) [18]	32, Male	Caecum and transverse colon	Toxic megacolon
Fischer et al. (2013) [19]	20, Male	Caecum	No toxic megacolon
Jaine et al. (2019) [3]	32, Male	Caecum	No toxic megacolon
Chu et al. (2022) [2]	15, Male	Sigmoid colon	No toxic megacolon
Kundoly and Jolly (2023) [20]	22, Male	NA	Granulomatous enteritis and intestinal obstruction
Current case	44, Female	Caecum	No toxic megacolon

## Conclusions

This case report presented a rare complication of *C. jejuni* infection leading to pancolitis and a caecal perforation requiring immediate surgical intervention in the form of emergency laparotomy and colectomy. Early recognition was vital for the successful management of such atypical complications. Therefore, in *C. jejuni* cases where hemodynamic compromise occurs with clinical worsening of symptoms despite adequate medical management, clinicians should commit to serial abdominal examinations with a low threshold or radiological imaging.
